# Acute promyelocytic leukaemia in a Nigerian patient—a case report depicting challenges in the management of haematological malignancies in Nigeria

**DOI:** 10.3332/ecancer.2025.1896

**Published:** 2025-04-23

**Authors:** Lemchukwu C Amaeshi, Yusuf Adelabu, Olufunto Kalejaiye

**Affiliations:** 1 Montefiore Medical Center, Bronx, NY 10467, USA; 2 College of Medicine University of Lagos, Lagos, Nigeria; 3 Lagos University Teaching Hospital, Lagos, Nigeria

**Keywords:** haematological malignancies, acute promyelocytic leukaemia, diagnostic delays, treatment delays, sub-Saharan Africa, Nigeria

## Abstract

**Introduction:**

Haematological malignancies (HMs) account for approximately 10% of all malignancies in sub-Saharan Africa (SSA), and their incidence is rapidly increasing. Successful management of these malignancies depends on early presentation, diagnosis and prompt initiation of treatment. However, in SSA and many low and middle-income countries, several barriers hinder the effective management of these malignancies. This case report of a patient with acute promyelocytic anaemia highlights the challenges in managing HM.

**Case summary:**

A 31-year-old woman presented to a primary health care centre with recurrent rectal bleeding and was diagnosed with haemorrhoids. She was given iron and was reassured of her symptoms. However, when her symptoms persisted, she was referred to an academic medical centre for definitive management of her haemorrhoids. On further evaluation, she was diagnosed with acute promyelocytic anaemia based on morphologic findings, as further morphological and molecular analysis could not be done due to the non-availability of advanced diagnostic infrastructure locally. Treatment initiation with all-trans-retinoic acid (ATRA) and arsenic trioxide (ATO) was delayed due to the unavailability of the medication locally and, therefore, had to be ordered from a different country. During this time, she developed febrile neutropenia and sepsis, and her care was limited by the unavailability of blood products and the unaffordability for necessary supportive medications. Eventually, she could only start ATRA as she could not afford ATO. As a result, she could not complete the entire course of treatment. Despite this, she showed clinical improvement and some haematological recovery and was discharged but, unfortunately, was lost to follow-up in the outpatient setting.

**Conclusion:**

Several barriers exist in managing HM and other cancers in general in SSA. Overcoming these barriers and improving outcomes in HM requires capacity building, international collaboration and political engagement.

## Background

Cancer has become a global epidemic [1, 2]. In sub-Saharan Africa (SSA), it is one of the leading causes of death and disability [3]. With the increasing incidence, cancer may replace infectious diseases as the number one cause of death [4]. GLOBOCAN 2020 reported more than one million cancer cases and over 700,000 cancer-related deaths, which represents an 85% increase from 2008 GLOBOCAN reports in the region [5]. Haematological malignancies (HMs), an emerging cause of significant morbidity and mortality, account for 10.8% of all malignancies in SSA and leukaemia accounts for 3.2% of this population [6, 7]. In Nigeria, hospital-based studies report that HM constitutes 17.4% to 18.0% of all cancers [8, 9].

Acute promyelocytic leukaemia (APL), a type of acute myeloid leukaemia, is characterised by a distinctive morphology of myeloid blasts and a specific reciprocal translocation [15,17], which fuses the promyelocyte gene on chromosome 15 to the retinoic acid receptor-α gene on chromosome 17 [10–12]. It can be fatal because of the risk of developing life-threatening disseminated intravascular coagulopathy (DIC). The discovery of all-trans-retinoic acid (ATRA) and arsenic trioxide (ATO) has revolutionised the treatment and prognosis of APL. In high-income countries, approximately 90%–95% of APL patients achieve complete remission (CR), attributed to the availability of prompt diagnostics and treatment for managing acute leukaemia and other HM [13]. However, in low and middle-income countries (LMICs), the inadequate skilled workforce, lack of diagnostic infrastructure and unavailability of appropriate treatment modalities pose significant challenges in managing HM [14]. This case report highlights some challenges we face in managing HM in Nigeria.

## Case summary

A 31-year-old Nigerian woman presented to the Accidents and Emergency Unit of an academic hospital in Lagos, Southwest Nigeria, with a month-long history of recurrent rectal bleeding. It initially started as stools mixed with blood, which progressed to passing frank bright red blood with blood clots, with an average of four episodes per day. There was associated with haemoptysis, bleeding from the gums, generalised weakness and dizziness. She denied having fever, abdominal pain, abdominal swelling, nausea, vomiting or diarrhoea. Earlier, she had visited a primary health care centre, where she was found to have haemorrhoids, thought to be the cause of her anaemia. She was reassured that the symptoms would improve and was given iron tablets. However, with the persistence of symptoms, she was referred to the academic hospital for definitive treatment of her haemorrhoids.

On physical examination, she had a subconjunctival haemorrhage, bleeding from the oral mucosa and frank blood on digital rectal examination. She was tachycardic with a pulse rate of 112 beats per minute, and her blood pressure was 154/94 mmHg. Her full blood count (FBC) revealed a haemoglobin (Hb) level of 6.1 g/dL; total leucocyte count, 1,360 cells/mm^3^; neutrophil count of 39.4 cells/mm^3^; lymphocytes count of 670 cells/mm^3^; monocytes: 570 cells/mm^3^; platelets: 8,000 cell/mm^3^. Peripheral blood film showed increased mononuclear cells with a high nuclear-cytoplasmic ratio, open chromatin and hyper-granular cytoplasm. Her prothrombin time (PT) and activated partial prothrombin time (APTT) were 10.6 and 31.4 seconds, respectively, and her international normalised ratio (INR) was 1.14. Urinalysis revealed significant microscopic haematuria. We checked hepatitis panels A, B and C, which were negative, and a preliminary autoimmune screen was negative. An abdominopelvic ultrasound scan was remarkable for splenomegaly. A bone marrow aspiration cytology revealed markedly hypercellular marrow with trails and marked infiltration by myeloblasts, mostly hypergranular, and features of reduced erythropoiesis and thrombopoiesis. The bone marrow biopsy sample was shipped overseas due to the unavailability of local expertise and resources for immunophenotyping, and we had to wait 14 days to obtain the result. The histology revealed a markedly diffuse hypercellular marrow with primitive cells and limited maturation to mature forms. The nuclei of the myeloblasts appeared markedly indented and folded with a high nucleocytoplasmic ratio. Unfortunately, cytogenetic analysis and molecular studies could not be performed because of their unavailability locally and the high cost of doing them abroad, which the patient could not afford as all the tests were paid for out-of-pocket, with no health insurance to cover the expenses.

Given the above findings, we made a presumptive diagnosis of low-risk APL (hypergranular variant) and decided to start treatment with ATRA and ATO. However, these medications were unavailable in the country, so they had to be ordered from overseas, resulting in an additional 6-week wait before treatment could commence. During her hospitalisation, we relied on providing supportive care until ATRA became available. She was transfused with several units of platelet concentrate and packed red blood cells as she had significant pancytopenia. Furthermore, there was a delay in obtaining blood products because of its limited availability at the blood bank. In most cases, her family members had to donate blood to the blood bank before blood products were released to transfuse the patient, and occasionally, it had to be sourced from a different hospital. She also developed febrile neutropenia with severe oesophageal candidiasis and was placed on meropenem and intravenous fluconazole. The high cost of purchasing these drugs led to times of medication nonadherence.

Eventually, ATRA was initiated. We planned to induce her with ATRA and ATO (ATRA 22.5 mg/m² PO twice daily from day 1 until hematologic CR or a maximum of 60 days and intravenous ATO at 0.15 mg/kg daily from day 1 until hematologic CR or a maximum of 60 days); however, she could not afford ATO. Therefore, she was treated with ATRA monotherapy (22.5 mg/m² PO twice daily until remission or a maximum of 90 days) [15]. She made significant improvement since the commencement of definitive treatment with ATRA until she was fit for discharge. Her FBC before discharge was: - Hb: - 8.66 g/dL, leucocyte count: -6,100 cells/mm^3^; neutrophils: 1,779 cells/mm^3^ lymphocytes: 1,834 cells/mm^3^; monocytes: 2,487 cells/mm^3^; platelets: −87,000 cell/mm^3^. She was followed up in the clinic for about 2 years, but unfortunately, we lost her to follow-up. Up until then, she had been off ATRA for at least 18 months because she could no longer afford the medication, but her blood count remained stable.

## Discussion

APL is a hematologic emergency that requires immediate intervention. When there is clinical suspicion and identification of APL-like features on a peripheral blood smear, ATRA should be initiated promptly to prevent life-threatening DIC and reduce early mortality. Diagnosis is confirmed through karyotyping, fluorescence *in situ* hybridisation (FISH) or polymerase chain reaction; however, treatment should not be delayed. Since its discovery in the late 1980s, ATRA has become a cornerstone of APL therapy. Risk stratification informs treatment selection. High-risk APL (WBC >10,000/µL) is associated with increased early mortality and thus necessitates chemotherapy, in addition to ATRA with or without ATO, to improve outcomes and reduce the risk of central nervous system infiltration [16]. Low-risk APL (WBC <10,000/µL) is managed with ATRA and ATO [17]. Supportive care is critical, especially for managing DIC, which includes daily monitoring of coagulation parameters (PT, aPTT, fibrinogen, fibrin degradation products) and maintaining fibrinogen at ≥100–150 mg/dL, platelets at >30–50 × 10⁹/L and INR <1.5. Supportive care also includes treating and preventing differentiation syndrome, a life-threatening complication of ATRA therapy, presenting with fever, dyspnea, pulmonary infiltrates, hypotension, and acute renal failure. It is commonly seen in high-risk APL, and management involves treating with corticosteroids and steroids prophylaxis recommended for patients with WBC >5–10 × 10⁹/L at presentation [18].

This case highlights the challenges of managing HM in a region with limited resources. Delays in diagnosis, late disease presentation, a dearth of trained haematologists, limited access to medication and the financial toxicity experienced by patients and their caregivers are some of the problems faced in treating HM in a low-resource setting such as ours.

Our patient had to wait till 1 month after the diagnosis was made before presenting to an academic hospital. Delays in diagnosis and referrals negatively affect the clinical outcome and prognosis of patients with HM. A literature review by Abel *et al* [19] on the delays in the referral and diagnosis of chronic HM showed that treatment delays start when a patient fails to see a healthcare provider on time and when providers fail to identify these malignancies and refer patients promptly. A study by Fasola et al [20] on the challenges of managing multiple myeloma in Nigeria revealed that more than 50% of patients experienced symptoms for more than 6 months before presenting to a healthcare provider. Because of the initially subtle presentation of most HM, there is a need to educate and increase the awareness of primary healthcare providers of these malignancies. Unfortunately, there is a scarcity of skilled manpower. According to the Africa Society for Blood Transfusion, there were only 70 trained haematologists in Nigeria, a country with a population of over 200 million [21]. This has been aggravated by physician shortages due to the ongoing brain drain of doctors and other healthcare workers [22].

The diagnosis of APL in our patient was made solely based on morphology. While HM can be diagnosed via basic stains such as haematoxylin and eosin, confirmation and disease characterisation require advanced diagnostic studies such as cytogenetic, molecular and FISH [23]. Unfortunately, in our case, these advanced studies were not possible due to the unavailability of the necessary infrastructure and the shortage of pathologists in the country. In SSA, there are only about 0.1 to 1.3 pathologists per million people, and most of them are concentrated in major cities. In 2019, Nigeria had only approximately 105 pathologists for a population of 200 million. In comparison, there were about 65 pathologists per million in the United States as far back as 2009 [24]. These shortages contribute to delays in diagnosis, ultimately leading to treatment delays and adverse clinical outcomes [25].

Patient-based factors such as socioeconomic and cultural factors also contribute to delays in diagnosing and treating HM. In a country where more than 70% of its citizens live under one dollar daily, most Nigerians pay out-of-pocket for their health bills [26]. Furthermore, for those with health insurance, which accounts for less than 5% of the population, it is not comprehensive to cover cancer care [27]. Usually, at the time of diagnosis, patients and their caregivers may be able to afford treatment, but this becomes unsustainable owing to the recurrent high costs of managing HM [28]. These patients and their families suffer considerable financial toxicity. With these prevailing problems, most patients will likely present to the hospital only when symptoms become intolerable. They will likely seek consultation with a local dispensary store staff, known locally as a ‘chemist’, where they are prescribed multiple analgesics for bone and generalised body pains or ‘blood tonics’ for ‘low blood levels.’ More worrisome is that owing to the subtle, nonspecific nature of these symptoms, they are instantly attributed to other diseases, such as malaria and typhoid fever, two commonly self-diagnosed medical conditions in the country [29,30]. Cultural factors also contribute, as these persistent symptoms are believed to be supernatural, exclusively treated through prayer and traditional/spiritual rites [31]. Sadly, by the time patients eventually present to the hospital, the disease has advanced. A study done in Jos, Nigeria, on the presentation and survival of patients with HM showed that 70% of patients presented with advanced stages of their disease [32].

The discovery of ATRA and ATO has revolutionised the management of APL, with the rate of achieving CR rate being as high as 90% to 95% [13]. ATRA and ATO have become the standard of care in patients with APL. However, these life-saving medications are not available in the country, and our patient had to wait 6 weeks before she could start any definitive treatment because the medications had to be imported from another country. Although it is concerning that such lifesaving medications are not readily available, most haematological chemotherapeutic drugs are expensive to procure, even for pharmacies in academic medical centres. With advances in treating hematologic malignancies in high-income countries and the shift to cellular therapy, these treatments are becoming the standard of care. However, the average Nigerian being treated for a HM may not have access to these advanced forms of treatment. We often rely on outdated chemotherapeutic regimens because they are less expensive and more affordable for the average Nigerian. However, they may not achieve the same efficacy as newer medications and may be associated with worse side effects.

Supportive care is crucial in managing HM due to the high risk of cancer-related complications and treatment-related toxicities. Patients may require close monitoring in an intensive care unit (ICU) due to these complications. However, this can be challenging due to the limited ICU bed capacity and high daily cost of ICU stays. This makes it difficult for patients and caregivers to afford [33]. Blood transfusion is fundamental in managing HM. Unfortunately, there is inadequate transfusion support due to the lack of a centralised national system for blood collection, coupled with a low whole blood donation rate, especially in SSA [34]. Instead, hospitals rely mainly on their local transfusion centres, which are frequently overburdened. According to the National Blood Service Commission, Nigeria only receives about 27% of its annual blood needs from voluntary blood donors, leaving a shortfall of about 73.3% yearly [35]. In most hospital systems, blood supplies are replenished through ‘replacement donations’ from family members or, in extreme cases, resort to commercial blood donors, which raises the risk of transmission of blood-borne infections [36]. Essential antimicrobials needed for treating severe infections may not be readily available, and even when they are, their costs can be prohibitive for patients and their families to afford. Finally, although we could not be sure why our patient was lost to follow-up, we could speculate that a contributing factor is the financial toxicity associated with her cancer care.

## Conclusion

In conclusion, this case underscores the difficulties that haematologists, patients and their caregivers encounter in managing HM in everyday practice, even at a tertiary level of health care. These challenges contribute to the high mortality and morbidity rates of HM in Nigeria and similar LMICs. Addressing these issues is no easy task, and a multipronged approach to finding solutions is paramount. Improving and building infrastructure to enhance the diagnosis of HM, raising community awareness of these cancers, building capacity to train and educate health care providers on the management of these cancers, fostering national and international collaboration and most importantly, engaging the government at all levels to make cancer care a significant priority. These efforts are necessary to mitigate the current challenges in caring for patients with HM.

## List of abbreviations

AML, Acute myeloid leukaemia; ANA, Anti-nuclear antibody; APL, Acute promyelocytic leukaemia; APTT, Activated partial prothrombin time; ATO, Arsenic trioxide; ATRA, All-trans-retinoic acid; CR, Complete remission; DIC, Disseminated intravascular coagulation; FBC, Full blood count; GLOBOCAN, Global Cancer Observatory; Hb, Haemoglobin; HM, Haematological malignancies; INR, International normalised ration; SSA, Sub-Saharan Africa.

## Conflicts of interest

There are no conflicts of interest to declare.

## Funding

No funding was received for this case report.

## Informed consent

Written consent was obtained from the patient.

## Author contributions

LC conceptualised and wrote the case report, while YA and OK edited and reviewed it.

## References

[1] Plummer M, Martel C, Vignat J (2016). Global burden of cancers attributable to infections in 2012: a synthetic analysis. Lancet Glob Health.

[2] World Health Organization DJ Global Health Estimates 2020: Deaths by Cause, Age, Sex, by Country and Region, 2000–2019.

[3] Gopal S, Wood WA, Lee SJ (2012). Meeting the challenge of hematologic malignancies in sub-Saharan Africa. Blood.

[4] https://www.afro.who.int/health-topics/noncommunicable-diseases.

[5] Sung H, Ferlay J, Siegel RL (2021). Global cancer statistics 2020: GLOBOCAN estimates of incidence and mortality worldwide for 36 cancers in 185 countries. CA Cancer J Clin.

[6] http://globocan.iarc.fr/Pages/fact_sheets_population.aspx.

[7] Okello CD, Niyonzima N, Ferraresso M (2021). Haematological malignancies in sub-Saharan Africa: east Africa as an example for improving care. Lancet Haematol.

[8] Nwannadi IA, Alao OO, Bazuaye GN (2011). The epidemiology of haematological malignancies at the University of Benin Teaching Hospital: a ten-year retrospective study. Int J Epidemiol.

[9] Akinde O, Anunobi C, Osunkalu O (2016). The challenges of lymphoma diagnosis in a Tertiary Hospital in Lagos, Nigeria. J Clin Sci.

[10] Bennett JM, Catovsky D, Daniel MT (1980). A variant form of hypergranular promyelocytic leukaemia (M3). Br J Haematol.

[11] Rowley JD, Golomb HM, Dougherty C (1977). 15/17 translocation, a consistent chromosomal change in acute promyelocytic leukaemia. Lancet.

[12] Grignani F, Ferrucci PF, Testa U (1993). The acute promyelocytic leukemia-specific PML-RAR alpha fusion protein inhibits differentiation and promotes survival of myeloid precursor cells. Cell.

[13] Wang ZY, Chen Z (2008). Acute promyelocytic leukemia: from highly fatal to highly curable. Blood.

[14] Akinde OR, Phillips AA, Oguntunde OA (2015). Cancer mortality pattern in Lagos University Teaching Hospital, Lagos, Nigeria. J Cancer Epidemiol.

[15] Tallman MS, Andersen JW, Schiffer CA (1997). All- trans -retinoic acid in acute promyelocytic leukemia. N Engl J Med.

[16] Iland HJ, Collins M, Bradstock K (2015). Use of arsenic trioxide in remission induction and consolidation therapy for acute promyelocytic leukaemia in the Australasian Leukaemia and Lymphoma Group (ALLG) APML4 study: a non-randomised phase 2 trial. Lancet Haematol.

[17] Lo-Coco F, Avvisati G, Vignetti M (2013). Retinoic acid and arsenic trioxide for acute promyelocytic leukemia. N Engl J Med.

[18] Sanz MA, Montesinos P (2014). How we prevent and treat differentiation syndrome in patients with acute promyelocytic leukemia. Blood.

[19] Abel GA, Friese CR, Magazu LS (2008). Delays in referral and diagnosis for chronic hematologic malignancies: a literature review. Leuk Lymphoma.

[20] Fasola F, Eteng K, Akinyemi JO (2012). Multiple myeloma: challenges of management in a developing country. J Med Med Sci.

[21] Chidebe RCW, Orjiakor TC, Lasebikan N (2023). Brain drain in cancer care: the shrinking clinical oncology workforce in Nigeria. JCO Glob Oncol.

[22] https://www.semanticscholar.org/paper/Physician-brain-drain-%3A-size-%2C-determinants-and-Docquier-Rapoport/2f741754ee67696ba66341f922cc2c5764b6c288#citing-papers.

[23] Snaith O, Poveda-Rogers C, Laczko D (2024). Cytogenetics and genomics of acute myeloid leukemia. Best Pract Res Clin Haematol.

[24] https://thepathologist.com/outside-the-lab/constant-demand-patchy-supply.

[25] Silas OA, Abdulkareem F, Novo JE (2022). Telepathology in Nigeria for global health collaboration. Ann Glob Health.

[26] World Bank Group Number of Poor Rate (Million) (%) National Poverty Line 71.0 46.0 2009 International Poverty Line.

[27] Awosusi A, Folaranmi T, Yates R (2015). Nigeria’s New Government and public financing for universal health coverage. Lancet Glob Health.

[28] Dieguez G, Ferro C, Rotter D (2018). The cost burden of blood cancer care A longitudinal analysis of commercially insured patients diagnosed with blood cancer.

[29] Enabulele O, Awunor SN (2016). Typhoid fever in a Tertiary Hospital in Nigeria: another look at the Widal agglutination test as a preferred option for diagnosis. Niger Med J.

[30] Odikamnoro OO, Ikeh IM, Okoh FN (2018). Incidence of malaria/typhoid co-infection among adult population in Unwana Community, Afikpo North Local Government Area, Ebonyi State, Southeastern Nigeria. Afr J Infect Dis.

[31] James PB, Asiimwe JB, Wardle J (2022). African culture, traditional medicine, and cancer care. Lancet Oncol.

[32] Egesie OJ, Jatau ED, Damulak OD (2017). Prevalence and type of haematological malignancies among adults in a tertiary hospital in Jos-Nigeria : a sixteen-year retrospective analysis. Highl Med Res J.

[33] Ogunbiyi O, Sanusi A, Osinaike B (2021). An overview of intensive care unit services in Nigeria. J Crit Care.

[34] Doughty HA, Hervig TA (2022). Whole blood for transfusion in sub-Saharan Africa. Lancet Glob Health.

[35] https://www.afro.who.int/countries/nigeria/news/blood-donation-selfless-life-saving-act.

[36] Nwogoh B, Ikpomwen OD, Isoa EM (2011). Donor blood procurement and the risk of transfusion transmissible viral infections in a tertiary health facility in South-South Nigeria. Niger Med J.

